# On Megaloblastic Degeneration

**Published:** 1912-01-13

**Authors:** Gordon R. Ward

**Affiliations:** West End Hospital for Nervous Diseases.


### ON MEGALOBLASTIC DEGENERATION.

By GORDON R. WARD, M.B. Lond., R.M.O., West End Hospital for Nervous Diseases.

This condition is considered by some to be diagnostic of anæmias which are essentially progressive and fatal. Herein lies its peculiar interest and the need for a right comprehension of its real significance.

### The Criterion.

The criterion of megaloblastic degeneration is the occurrence of megaloblasts in the bone-marrow of adults. Normally they are not to be found after two years of age. These megaloblasts are large nucleated red corpuscles with a particular type of nucleus which is not difficult to recognise. The following quotation is from the well-known work on anæmia of Ehrlich and Lazarus, a work which first drew attention to the alleged " pernicious " significance of this change: " All attempts to smooth over the differences between megaloblasts and normoblasts, or to deny that such differences exist, must fail before the clinical fact that the blood of pernicious anæmia is a megalocytic blood. The appearance of megaloblasts and megalocytes is a proof that the regeneration of the blood in the bone-marrow does not take place normally." The pernicious character may be regarded as sufficiently proved " if a considerable portion of the bone-marrow, and not necessarily the whole of it, reveals megaloblastic degeneration." The term " pernicious " anæmia used here avowedly identified Ehrlich's group with Biermer's classification of anæmias, made purely on a clinical basis. The chief point of this clinical basis was the " complication with fatty degeneration of the heart and vessels."

It is my desire, accepting Ehrlich's definition of megaloblastic degeneration, to show that the term " pernicious " is quite inapplicable to such cases, since many of them are secondary to removable causes; and further to demonstrate that, however interesting megaloblastic degeneration may be to the pathologist, it is a most untrustworthy guide to the physician. Since it is seldom possible to examine the bone-marrow of living persons, this " degeneration " must be judged of by the presence of megaloblasts and megalocytes in the circulating blood. In the following cases (again accepting Ehrlich's definition) the occurrence of even one megaloblast is considered proof of the " degeneration," while the presence of 20 per cent. or more megalocytes is taken to indicate the involvement of " a considerable portion of the bone-marrow." Only those cells which are thoroughly typical have been included as megaloblasts, and 9 $\mu$ is taken as the size below which cells may not be called megalocytes. The longest diameter of cells has been measured, but poikilocytes are not included.

### Two Cases.

The following are two cases in which only one megaloblast was seen:—

Case I.—C. I., aged 51. R.B.C.=2,500,000 per c.mm. Hæmoglobin=74 per cent. Colour index=1.48. This was a typical case of Addisonian anæmia. The patient was a military man of exceptional mental ability, and he described most clearly the initial onset of soreness of the tongue. He had " recovered " once, and the second attack was preceded by the same soreness. His colour was typical, but no estimation of urobilin was made. When the above count was made he was issuing satisfactorily from his second attack.

Case II.—W. M., aged 63. R.B.C.=3,750,000 per c.mm. Hæmoglobin=96 per cent. Colour index=1.28. The patient was a labourer, and was admitted to hospital for acute abdominal disease. He was found to be suffering from pericarditis, and made a slow but uneventful recovery. He had no lemon colour or glossitis. Four days previous to the above examination a count had shown R.B.C. = 7,062,000 per c.mm. Hæmoglobin=114 per cent. Colour index=0.814. This was taken as evidence of concentration due to heart failure, and the later count followed the administration of drugs which corrected this condition.

### The Deduction.

It is perfectly obvious that if such cases as these are to be grouped together on the strength of the presence in each of one megaloblast, the physician at least should disregard the grouping, for it can be no help to him, and can afford no hope to the patient.

Case III.—M. D., adult. R.B.C.=820,000 per c.mm. Hæmoglobin=24 per cent. Colour index=1.46. Megaloblasts=88 per c.mm. Again a typical case of Addisonian anæmia. The patient was almost moribund when this count was done. Twenty-four hours later she was sitting up and demanding food. This sort of thing does not occur in other anæmias. She left hospital, but a relapse later proved fatal.

Case IV.—X. Y., adult. R.B.C.=4,760,000 per c.mm. Hæmoglobin=65 per cent. Colour index=0.68. Megaloblasts=98 per c.mm. This patient had had one breast removed for malignant disease, and had recurrence in the scar and involving the sternum and two ribs. She died about two weeks after this count was done.

In these two cases the degree of anæmia was very different, but the number of megaloblasts was similar. Had the recurrent carcinoma been in some position not very obvious clinically, *e.g.* the spine or long bones, in the absence of spontaneous fracture it might have been very difficult to avoid making a mistake in diagnosis; if the megaloblasts were held to have the significance attributed to them by the Biermer-Ehrlich theory, a mistake was certain.

### Typical Cases.

The next case is very similar to Case V., and shows that the number of megaloblasts is of no clinical significance.

Case V.—M. P., aged 44. R.B.C.=4,920,000 per c.mm. Hæmoglobin=50 per cent. Colour index = 0.51 Megaloblasts = 2 per c.mm. This was a case of recurrent carcinoma in the spine following removal of the breast.

In spleno-medullary leukæmia it is very common to find megaloblasts—rather the rule than the exception. Case VI. is a typical case.

Case VI.—J. T., aged 48. Admitted to hospital for distension of abdomen—found to be due to large spleen. On three consecutive occasions the megaloblasts were found to be respectively 456, 260, and 99 per c.mm. He was undergoing $x$-ray treatment, and these counts were done at intervals of five days. Some of these megaloblasts were as large as 20 $\mu$, the largest ones being those that showed mitosis.

### Unreliable Criteria.

But if the actual presence of megaloblasts in different cases demonstrates that they are quite unreliable as criteria of any particular variety of anæmia, the occurrence of megalocytes affords even more striking proof of the same view. Cases VII. to XI. show in how wide a variety of conditions more than 20 per cent. of the red cells may be megalocytes.

Case VII.—J. P., aged 15. R.B.C. = 2,860,000 per c.mm. Hæmoglobin = 56 per cent. Colour index = 1.0. Megalocytes = 28 per cent. Average size of R.B.C. = 7.81 $\mu$. Largest megalocytes = 11 $\mu$. A case of tuberculosis with moderate involvement of the right apex. He made a good recovery from the anæmia, and the tuberculosis also was improved by sanatorium treatment.

Case VIII.—E. L., aged 17. R.B.C. = 2,800,000. Hæmoglobin = 30 per cent. Colour index = 0.535. Megalocytes = 21 per cent., the largest measuring 11 $\mu$. Average size of corpuscles = 7.5 $\mu$. This was a typical case of chlorosis. Three weeks later the R.B.C. = 5,787,000 per c.mm.; the hæmoglobin, as is usual in these cases, was still comparatively low, viz. 58 per cent.

Case IX.—H. H., aged 8. R.B.C. = 3,650,000. Hæmoglobin = 80 per cent. Colour index = 1.09. Megalocytes = 53 per cent., the largest measuring 11 $\mu$. The average size of R.B.C. = 8.68 $\mu$.

This count was done five weeks after the onset of an attack of measles. The patient had been much neglected, and was suffering from starvation. She made an excellent recovery when placed under suitable conditions. No physical signs or symptoms of any organic disease appeared at any time.

### A Case of Septicæmia.

Case X.—E. M., aged 46. R.B.C. = 3,487,000. Hæmoglobin = 60 per cent. Colour index = 0.87. Megalocytes = 26 per cent., the largest measuring 11 $\mu$. The average size of R.B.C. = 8.02 $\mu$.

This was a case of septicæmia, originating in the left fallopian tube, but at the time of examination the liver was almost wholly occupied with abscesses of various sizes. Streptococci in pure culture were isolated—indeed, even the small piece of tissue which came away in the end of an exploring needle was full of them. The patient died a few days later.

Case XI.—G. Q., aged 62. R.B.C. = 920,000 per c.mm. Hæmoglobin = 25 per cent. Colour index = 1.39. Megalocytes = 62 per cent., the largest measuring 12 $\mu$. The average size of R.B.C. = 8.8 $\mu$.

No nucleated R.B.C. were seen.

This was a case of septic anæmia also, but the sepsis was confined to the gastro-intestinal tract. The writer accidentally pricked his finger while removing the tongue at autopsy. This was at four o'clock in the afternoon. He was wakened up by pain in the finger at about four o'clock the next morning, so that there can be little doubt that virulent organisms were present. The case was complicated by severe epistaxis. It had been diagnosed as Addisonian anæmia about a month before, but this was disproved by the fact that the marrow was found atrophic and pale, and the spleen bloodless; nor was there a typical glossitis.

### Why These Cases are Valuable.

These cases were not collected with a view to their publication in this form, so that the personal factor in the above measurements, etc., is, as far as possible, eliminated. The diseases in which the presence of megalocytes is occasional are so numerous that they probably include nearly every anæmia of severity. The following may be mentioned from my own records:—

| | |
|---|---|
| Acne ... ... ... ... ... | R.B.C. = 6,590,000 |
| Cholæmia ... ... ... ... | R.B.C. = 2,958,000 |
| Distomiasis ... ... ... ... | R.B.C. = 4,700,000 |
| Erythæma ... ... ... ... | R.B.C. = 10,480,000 |
| General Paralysis ... ... ... | R.B.C. = 5,380,000 |
| Intestinal Obstruction ... ... | R.B.C. = 6,025,000 |
| Lymphæmia ... ... ... ... | R.B.C. = 1,530,000 |
| Osteo-myelitis ... ... ... | R.B.C. = 7,300,000 |
| Periodic Insanity ... ... ... | R.B.C. = 3,741,000 |
| Traumatic Anæmia ... ... | R.B.C. = 4,005,000 |
| Sarcoma ... ... ... ... | R.B.C. = 1,875,000 |

It is also apparent from this list, which represents a very small number of the cases which might be quoted, that it is not necessary that there should be any great degree of anæmia before this megaloblastic "degeneration" occurs. None of these cases were in children.

### An Inapplicable Term.

No one reading even the above short records can possibly, one ventures to think, suppose that the term "pernicious" is in any way applicable to megaloblastic anæmias; or that this hæmatological change bears any relation to ætiology, prognosis, or treatment. What its precise significance may be we are at present unaware. There can be no doubt that it is more constantly associated with Addisonian anæmia than with other varieties, but at the same time it is not in every case or at all times associated with this condition, and is most certainly not diagnostic of it. In the same way are poikilocytosis and a high colour index in no way pathognomonic of any one clinical variety of anæmia, and I could adduce another series of cases in support of this statement; but at present it is sufficient if this fallacy of supposing that megaloblastic degeneration has a clinical significance of its own apart from other signs and symptoms is made apparent.

